# Laboratory Infection of Novel Akhmeta Virus in CAST/EiJ Mice

**DOI:** 10.3390/v12121416

**Published:** 2020-12-09

**Authors:** Clint N. Morgan, Audrey M. Matheny, Yoshinori J. Nakazawa, Chantal Kling, Nadia Gallardo-Romero, Laurie Seigler, Galileu Barbosa Costa, Christina Hutson, Giorgi Maghlakelidze, Victoria Olson, Jeffrey B. Doty

**Affiliations:** 1 Poxvirus & Rabies Branch, National Center for Emerging Zoonotic Infectious Diseases, Centers for Disease Control and Prevention, Division of High-Consequence Pathogens & Pathology, Atlanta, GA 30329, USA; nvs8@cdc.gov (A.M.M.); inp7@cdc.gov (Y.J.N.); lwu4@cdc.gov (C.K.); hfa5@cdc.gov (N.G.-R.); ogq1@cdc.gov (L.S.); zuu6@cdc.gov (C.H.); vao9@cdc.gov (V.O.); uwb7@cdc.gov (J.B.D.); 2 Oak Ridge Institute for Science and Education, CDC Fellowship Program, Oak Ridge, TN 37830, USA; 3 Kāpili Services, LLC, An Alaka’ina Foundation Company, Honolulu, HI 96814, USA; 4 Núcleo de Epidemiologia e Bioestatística, Centro de Pesquisas Gonçalo Moniz, Fiocruz, Bahia 40296-710, Brazil; galileuk1@gmail.com; 5 U.S. Centers for Disease Control and Prevention, South Caucuses Office, Tbilisi 0177, Georgia; kuo9@cdc.gov

**Keywords:** novel *Orthopoxvirus*, zoonosis, *Mus musculus*, pathogenesis

## Abstract

Akhmeta virus is a zoonotic *Orthopoxvirus* first identified in 2013 in the country of Georgia. Subsequent ecological investigations in Georgia have found evidence that this virus is widespread in its geographic distribution within the country and in its host-range, with rodents likely involved in its circulation in the wild. Yet, little is known about the pathogenicity of this virus in rodents. We conducted the first laboratory infection of Akhmeta virus in CAST/EiJ *Mus musculus* to further characterize this novel virus. We found a dose-dependent effect on mortality and weight loss (*p* < 0.05). Anti-orthopoxvirus antibodies were detected in the second- and third-highest dose groups (5 × 10^4^ pfu and 3 × 10^2^ pfu) at euthanasia by day 10, and day 14 post-infection, respectively. Anti-orthopoxvirus antibodies were not detected in the highest dose group (3 × 10^6^ pfu), which were euthanized at day 7 post-infection and had high viral load in tissues, suggesting they succumbed to disease prior to mounting an effective immune response. In order of highest burden, viable virus was detected in the nostril, lung, tail, liver and spleen. All individuals tested in the highest dose groups were DNAemic. Akhmeta virus was highly pathogenic in CAST/EiJ *Mus musculus,* causing 100% mortality when ≥3 × 10^2^ pfu was administered.

## 1. Introduction

Within the family *Poxviridae*, most extant members of the genus *Orthopoxvirus* (OPXV) are zoonotic, with broad mammalian host ranges or host systems that remain cryptic [1]. The primary exception to this ‘rule’ is *Variola virus* (the causative agent of smallpox), which was highly specialized as a human-only pathogen, making its eradication possible following immense global public health vaccination campaigns [2]. In recent years, new potential mammalian hosts of OPXVs have been identified [3,4,5,6,7] and an increasing number of novel OPXVs are being detected, including *Akhmeta virus* (AKMV) from the country of Georgia [8,9,10,11,12].

AKMV was first identified in 2013 after two men in the country of Georgia were exposed to the virus through contact with infected cattle, and subsequently developed cutaneous lesions [12]. During the outbreak investigation, 59.5% (*n* = 37) of humans, 100% (*n* = 11) of the herd cared for by the index patients, and 4.2% (*n* = 24) of a nearby herd were positive for anti-OPXV antibodies. Additionally, 35.3% (*n* = 34) of the small mammals collected in the same area showed IgG antibodies against OPXVs. Beginning in 2015, ecological investigations were conducted in several regions of Georgia to understand the circulation of the virus in its natural hosts/reservoirs [5]. These investigations suggest the virus has a broad geographic distribution in Georgia and have yielded five viral isolates from lesion and heart/lung material collected from sylvatic rodents of the genus *Apodemus* [5].

The primary goal of this infection study was to gather baseline data on pathogenicity, antibody response, and tissue tropism of the novel AKMV in the house mouse (*Mus musculus*). CAST/EiJ mice (abbreviated as CAST) are originally derived from wild mice collected in Thailand and are often bred with the common laboratory strains to generate F1 hybrids with high levels of heterozygosity. CAST mice share high genetic similarity with wild *Mus musculus*, and were selected in an effort to characterize this virus in commercially available rodents that most resemble those that may serve as natural reservoirs. 

## 2. Materials and Methods

### 2.1. Animals and Experimental Groups

For this study, 30 adult female CAST mice were acquired from the Jackson Laboratory (Stock No. 000735) and brought into a Centers for Disease Control and Prevention (CDC) Biosafety Level 2 animal facility in June of 2018, with animal work and sampling performed using Biosafety Level 3 practices. The 30 mice were divided into six experimental groups of five animals each. One group served as a negative control, receiving vehicle only (PBS + 0.05% fetal bovine albumin; FBA). The other five groups received infectious doses of <1, 2, 10^2^, 10^4^ and 10^6^ plaque forming units (pfu), respectively, verified by back-titration. During the study, the animals were inspected for presence of skin lesions, overall health and general body condition, and weighed three times per week. This study was approved by the Animal Care and Use Committee of the CDC, under protocol number 2789DOTMOUC-A1.

### 2.2. Sampling Regimen

Animals were handled under anesthesia with 1–5% isoflurane gas via induction chamber or nasal cone. Prior to infection, blood was collected from the submandibular vein via cheek puncture using a sterile 5.5 mm lancet (Medipoint, Inc., Mineola, NY, USA), and an oral swab was collected. Throughout the study, oral and anal swabs were collected three times per week. A dried blood sample was collected from the submandibular vein on Nobuto filter paper (Advantec, Tokyo, Japan) weekly at day 7, 14, 21, 28, and 35 post-infection. A pain score was used to determine euthanasia criteria for animals based on various clinical signs of infection and weight loss (Table 1). If euthanasia criteria were met, oral and anal swabs were collected, followed by exsanguination via intracardiac puncture under deep anesthesia with 5% isoflurane gas, and then cervical dislocation. The blood collected was prioritized for serology and collected in a 2 mL serum separator tube. If sufficient blood volume was collected, an aliquot of blood was also collected in a 2 mL EDTA (anticoagulant for whole blood analysis) tube for real-time polymerase chain reaction (PCR) testing and viral titration. Following euthanasia, a necropsy was performed using aseptic techniques and the following tissue samples were collected and placed in 2 mL O-ring sealed cryotubes: nostrils (external nares), heart, lung, liver, spleen, kidney, gonad, and a tail clip. Tissue samples, EDTA whole blood, and swabs were stored at −80 °C prior to sample processing. The blood samples in serum separator tubes were centrifugated to obtain blood sera. Sera and dried blood samples were tested by Enzyme-Linked Immunosorbent Assay (ELISA) to assess immune response. Tissue, whole blood and swab samples were processed and screened for presence of viral deoxyribonucleic acid (DNA) by PCR, and viral load was assessed by viral titration in cell culture. The AKMV-specific PCR assay used in this study was performed as described in Doty et al., 2019.

### 2.3. Inoculation and Back-Titration

Under anesthesia with 1–5% isoflurane gas via induction chamber, animals were inoculated with AKMV intranasally with 10 µL (5 µL per nostril) of the designated target viral dose diluted in phosphate buffered saline (PBS; 7.4 pH) + 0.01% of FBA. The stock AKMV virus used for inoculum in this study was first isolated from a human in the country of Georgia during the 2013 outbreak investigation [12]. Immediately following the inoculation, the inocula underwent viral titration to confirm the titer of each dose administered to the groups. Six-well plates with a confluent monolayer of BSC-40 cells were used to titer each inoculum. Duplicate wells were inoculated with 10-fold dilutions of the inocula used for the experimental groups. Infected cells were incubated at 35.5 °C and 6% CO_2_, in Roswell Park Memorial Institute medium (RPMI) with 2% fetal bovine serum, 1% of 100X penicillin/streptomycin, and 1% of 200 mM L-glutamine (RPMI 1640; CDC, BIOS Product ID #11880). Cells were monitored for characteristic signs of cytopathic effect via microscopy. At 72 h post inoculation, cells were stained with 2X formalinized crystal violet stain (CDC, BIOS Product ID #7121), and plaques were enumerated to determine the actual viral titer per animal group.

### 2.4. Sample Processing and DNA Extraction

Swabs were hydrated in 400 µL of sterile PBS (pH 7.4) for five minutes at room temperature and transferred to swab extraction tube system (SETS; Cat. No. 3315568, Roche, IN, USA) tubes. SETS tubes were centrifuged at 6000 rpm for one minute to collect the elute, after which, inner SETS tubes and swabs were discarded. Tissues were homogenized in 500 µL of PBS with a sterile steel bead in two three-minute runs (icing in between) using a Geno Grinder 2000 (SPEX SamplePrep LLC, Metuchen, NJ, USA). A total of 100 µL of either swab eluate or homogenized tissue were aliquoted and used for DNA extraction; the remaining eluate or tissue homogenate volume was kept for viral titration. DNA was extracted from all swab and tissue samples using the MagMAX DNA Multi-sample Ultra kit (Applied Biosystems, Foster City, CA, USA) extraction protocol on a MagMAX Express-96 deep well magnetic particle processor (Applied Biosystems).

### 2.5. PCR

All samples were screened for presence of AKMV DNA by real-time PCR using the AKMV specific primers and probe described in Doty et al., 2019 on the VIIA7 real-time PCR system (Applied Biosystems) with the following run conditions: one cycle of 95 °C for 10 s followed by 40 cycles of 95 °C for 1 s and 60 °C for 20 s. Each sample was run in duplicate with an AKMV DNA positive control included on every plate. Samples were considered positive by PCR if both wells showed amplification and the average threshold cycle (C_t_) value for sample duplicates was <37.0. Samples with inconclusive results (average C_t_ values 37.0–39.9 or discrepant results between duplicates) were tested again and the subsequent results (if conclusive) replaced the preliminary inconclusive testing values. After re-analysis, samples with average C_t_ values > 37.0 or discrepant duplicate results were considered negative for presence of AKMV DNA. Samples positive for presence of AKMV DNA by real-time PCR were selected for viral titration to determine presence and quantification of viable virus.

### 2.6. Viral Titration

Following PCR screening, and if positive, viral burden in tissue, swab, and whole blood samples was evaluated via tissue culture and viral titration following similar methodology as described in the related literature [13]. A total of 150 µL of swab eluate or tissue homogenate were serial diluted in 2% RPMI medium, and 650 µL of each dilution were added to 6-well plates in duplicate on BSC-40 cell monolayers. These plates were then incubated at 35.5 °C and 6% CO_2_ for approximately 72 h, then 2X formalinized crystal violet stain was added to each well to inactivate the virus and facilitate the enumeration of the viral plaques. Titers are expressed as pfu per milliliter (pfu/mL).

### 2.7. Serologic Testing

Serum samples were tested via ELISA for the detection of OPXV immunoglobulin G antibodies as described by Gallardo-Romero et al., 2016. A crude preparation of the Western Reserve vaccinia virus strain was used at a concentration of 0.01 µg/well, diluted in carbonate buffer (carbonate–bicarbonate buffer, Sigma-Aldrich Corporation, St. Louis, MI, USA; pH 9.6), and used to coat half of the wells of each microtiter plate. The other half of each microtiter plate was coated with 0.01 µg/well of BSC-40 cell lysate (CDC Core Facility, Atlanta, GA, USA). Animal sera were tested in duplicate at 1:100 dilution. The average of the OD values from the duplicate of a sample in the virus half of the plate minus the average plus 2 SDs of the duplicates from the same sample in the cell lysate half of the plate was used to generate a cutoff value. A sample was considered positive by ELISA for the presence of anti-OPXV antibodies if the average of the duplicates of the OD values was above the cutoff value.

### 2.8. Statistical Analysis

Statistical significance among the groups for dose-dependent effects on mortality, change in weight, and tissue-specific viral titer (pfu), were assessed with the non-parametric Kruskal–Wallis test. Dose-dependent effects on mortality were calculated among all groups, using the day post-infection an individual was euthanized due to clinical score. Weight loss among all groups was calculated as an individual’s percent weight change from the day of challenge to the day of euthanasia. Tissue-specific variation in viral burden among groups was tested for statistical significance. Analyses were performed in the program R v.3.6.3 (http://www.R-project.org/).

## 3. Results

### 3.1. Clinical Signs and Mortality

**Figure 1:**
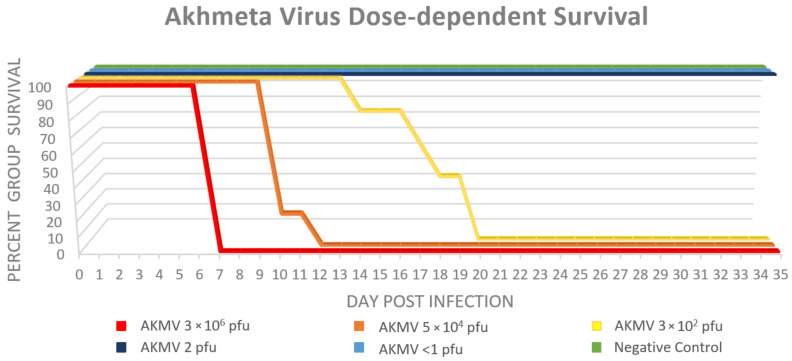
Survival curve by dose, of CAST/EiJ mice infected with Akhmeta virus.

**Figure 2:**
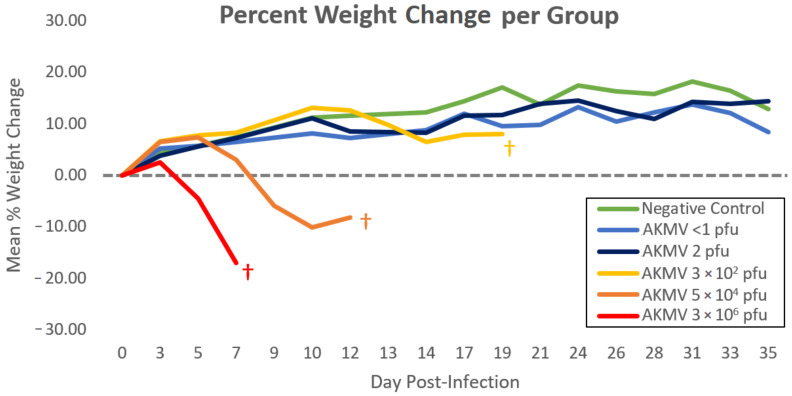
Mean percent weight change of each experimental group throughout the course of the experiment. Weight loss among all groups was calculated as an individual’s percent weight change from the day of challenge to the day of euthanasia. † = Termination of last individual in group due to clinical score ≥ 10.

No mortality was observed in the <1 pfu, or the 2 × 10^0^ pfu dose groups. All CAST mice in the three highest-dose groups demonstrated high morbidity and mortality after infection, with survival rates of 0%. A statistically significant (*p* = 2.5 × 10^−5^) dose effect was observed among the groups, with the 3 × 10^2^ pfu group requiring euthanasia at day 14–20, the 5 × 10^4^ pfu group at day 10–12, and the 3 × 10^6^ pfu all on day 7 post infection (Figure 1.). No animals were found dead as a result of succumbing to the infection; all were euthanized after reaching the maximum clinical pain score allowed (≥10) or, for animals in the two lowest dose groups, at the termination of the study. The most observed clinical sign of infection was piloerection (*n* = 15), followed by weight loss (*n* = 14) and labored breathing under anesthesia (*n* = 11). Other clinical signs were observed and tabulated in Table 2. The percent change in weight from day 0 to euthanasia was significantly different among all groups (*p* = 5.1 × 10^−4^). The moving average of percent weight change is displayed in Figure 2.

### 3.2. PCR: Tissues, Whole Blood, and Swabs

Tissues collected from the negative control group were all negative, with the exception of one individual (M2) tail sample (C_t_ = 33.7). Within the AKMV < 1 pfu group only two tail samples (average C_t_ = 35.2) and one nostril sample (C_t_ = 35.3) were PCR-positive; no internal organs demonstrated quantifiable viral DNA. In the AKMV 2 × 10^0^ pfu group, two tail samples (average C_t_ = 33.6), one nostril sample (C_t_ = 33.5) and one spleen sample (C_t_ = 35.1) were PCR positive. In the three high-dose groups (AKMV 3 × 10^2^ pfu, 5 × 10^4^ pfu, and 3 × 10^6^ pfu), all individuals (*n* = 15) had PCR-positive tail, nostril, kidney, and liver samples; 14 individuals had PCR positive spleen and heart samples; 10 individuals had PCR positive lung samples; and 5 had PCR positive gonad samples. Within the three high-dose groups, the earliest C_t_ values were obtained from the nostril samples (average C_t_ = 17.2, *n* = 15), followed by tail (average C_t_ = 28.9, *n* = 15), liver (average C_t_ = 29.3, *n* = 15), and lung (average C_t_ = 33.1, *n* = 10).

Whole blood collected from the negative control group, AKMV < 1 pfu, and 2 × 10^0^ pfu groups were all negative by PCR. In the AKMV 5 × 10^4^ pfu group, two individuals were not tested due to insufficient blood volume at euthanasia; the remaining three individuals were all PCR-positive (average C_t_ = 30.5). The AKMV 5 × 10^4^ pfu group had one individual that was not tested due to insufficient blood volume at euthanasia; the remaining all were PCR-positive (average C_t_ = 28.5). Due to severe dehydration at euthanasia of all individuals in the AKMV 3 × 10^6^ pfu group, there was insufficient blood volume to use for assessment of DNAemia. 

**Figure 3:**
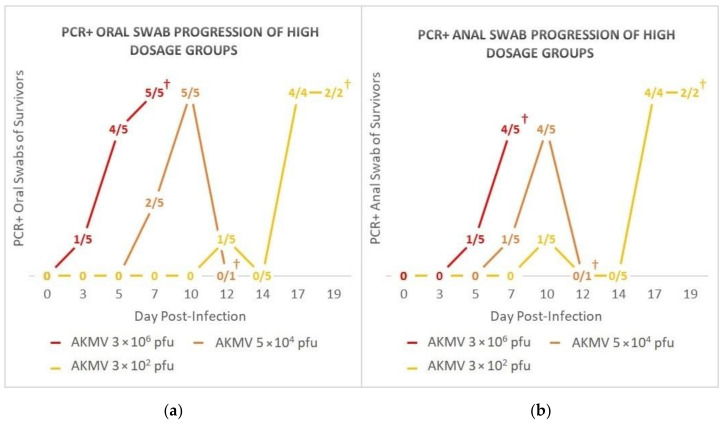
Progression of PCR-positive (**a**) oral swabs and (**b**) anal swabs over the course of infection. X/Y = number of PCR-positive swabs/number of animals remaining in group. † = Termination of last individual in group due to clinical score ≥ 10.

All oral and anal swabs collected from individuals in the negative control and AKMV 2 × 10^0^ pfu groups were negative by PCR. One oral swab from an individual on DPI 12 in the AKMV <1 pfu group was PCR-positive (C_t_ = 36.5); all anal swabs from this group were negative. PCR-positive oral and anal swabs of animals in the three highest dose groups followed a temporal dose-dependent progression (Figure 3). The presence of DNA was observed earliest in the oral and anal swabs of the 3 × 10^6^ pfu group (DPI 3 and DPI 5, respectively), and latest in the 3 × 10^2^ pfu group (DPI 12 and DPI 10, respectively). PCR-positive oral swabs were observed in the 3 × 10^2^ pfu group from DPI 12–19, in the 5 × 10^4^ pfu group from DPI 7–10, and in the 3 × 10^6^ pfu group from DPI 3–7. PCR-positive anal swabs were observed in the 3 × 10^2^ pfu group from DPI 10–19, in the 5 × 10^4^ pfu group from DPI 7–10, and in the 3 × 10^6^ pfu group from DPI 5–7. Overall C_t_ values of PCR positive swabs ranged from 30.2 to 37.0, and anal swab C_t_ values ranged from 25.9 to 37.0.

### 3.3. Viral Titration: Tissues, Whole Blood and Swabs

**Figure 4:**
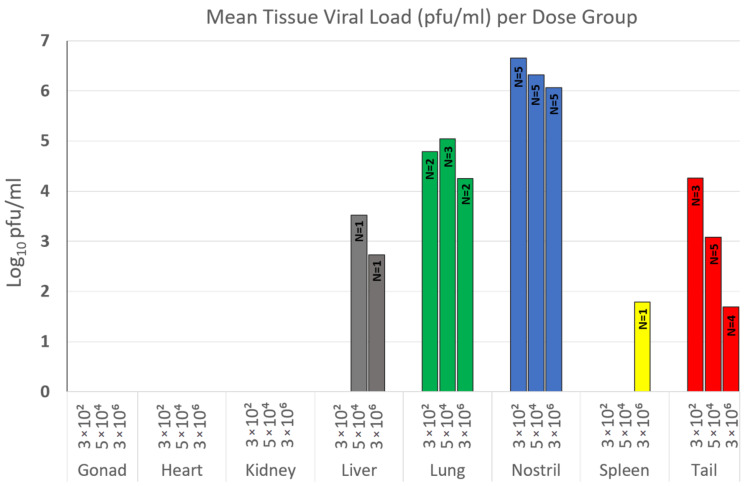
Mean viral loads of tissue samples within the three Akhmeta virus (AKMV) infected high-dose groups (3 × 10^2^ pfu, 5 × 10^4^ pfu, and 3 × 10^6^ pfu).

The one PCR-positive tail sample in the negative control group, and PCR-positive samples from the AKMV < 1 pfu group had no viable virus. The PCR-positive nostril sample from one individual (M12) in the AKMV 2 × 10^0^ pfu group had a viral load of 354 pfu/mL; no viable virus was detected in the other PCR-positive samples from this group. Among the three high-dose groups, all PCR-positive tissues were titrated (*n* = 85), and viable virus was detected only in nostril (average = 2.59 × 10^6^ pfu/mL, *n* = 15/15), tail (average = 5.11 × 10^3^ pfu/mL, *n* = 12/15), lung (average = 6.97 × 10^4^ pfu/mL, *n* = 7/10), liver (average = 1.92 × 10^3^ pfu/mL, *n* = 2/15), and spleen (6.2 × 10^1^ pfu/mL, *n* = 1/15) samples (Figure 4). Among all individuals in the high-dose groups, the viral loads of nostril samples ranged from 3 × 10^3^ to 2.08 × 10^7^ pfu/mL. Viral loads of tail samples ranged from 3.1 × 10^1^ to 5.46 × 10^4^ pfu/mL, and lung samples ranged from 1.46 × 10^2^ to 3.29 × 10^5^ pfu/mL. Since viable virus was only detected in multiple individuals from the three highest dose groups, only those groups were used for statistical comparisons in tissue-specific viral loads and there was no statistically significant difference among groups (*p* > 0.05).

The viral burden of all PCR-positive whole blood samples (*n* = 7), and oral and anal swabs (*n* = 47) was assessed in cell culture, and all were below the detectable limits of the assay.

### 3.4. Serology

**Figure 5:**
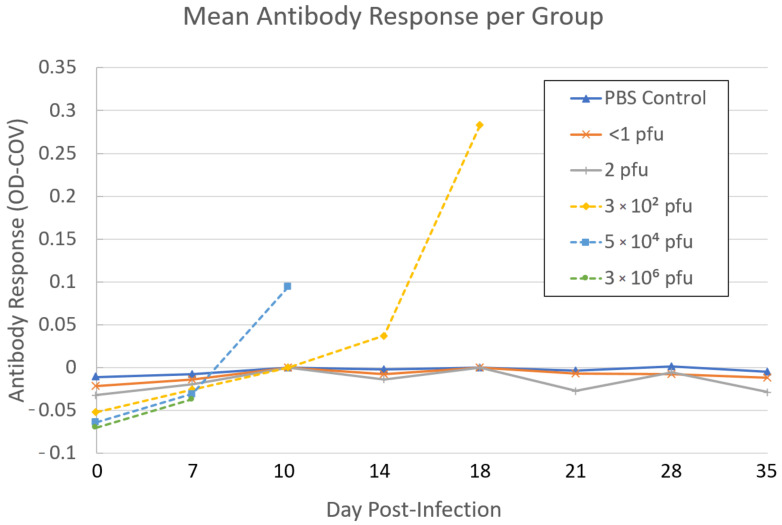
Mean antibody response of Cast/EiJ mice of all experimental groups.

All serum samples within the negative control group, <1 pfu group, and 2 × 10^0^ pfu group had no detectable anti-OPXV antibodies by ELISA. All five animals in the 3 × 10^2^ pfu group developed detectable antibodies beginning at DPI 14–20 until euthanasia, and overall OD-COV values ranged from 0.021 to 0.407 (Figure 5). The 5 × 10^4^ pfu group developed detectable antibodies beginning at DPI 10–12 until euthanasia, and overall OD-COV values ranged from 0.016 to 0.228 (Figure 5). Within the highest dose group, 3 × 10^6^ pfu, euthanasia of all individuals in this group occurred earlier than expected (DPI 7) and antibodies were not detected from the terminal blood collection. Across all animals with multiple blood collections, anti-OPXV antibody levels increased throughout the course of infection with highest values at euthanasia. Overall, following infection with AKMV, anti-OPXV antibodies were first detectable at 10 DPI with the assay utilized for this study.

## 4. Discussion

The intranasal route of infection was chosen to best compare our results with other similar studies of OPXV intranasal infections in CAST mice, and to mimic the upper respiratory transmission of OPXVs [14]. Similar patterns of pathogenicity have been observed with other OPXVs such as *Monkeypox virus*, *Cowpox virus*, and *Vaccinia virus* [15,16,17]. Infectious doses of AKMV in CAST mice are similar to intranasal infection of other OPXVs, for example, the LD_50_ of *Monkeypox virus* has been reported as 6.8x10^2^ pfu [15], and the LD_50_ of the Western Reserve strain of *Vaccinia virus* as well as the Brighton Red strain of *Cowpox virus* have both been reported as 1 × 10^2^ pfu [16]. As significant morbidity occurred following infection with AKMV at 3 × 10^2^ pfu (100% of animals were euthanized due to pain score by DPI 20), an LD_50_ could not be calculated in this study. It would be of interest to conduct additional experiments using multiple routes of infection (e.g., percutaneous); however, this is not within the scope of this study.

All individuals within the 3 × 10^6^ group were euthanized on the same day, individuals within the 5 × 10^4^ pfu group were euthanized within 48 h, and individuals in the 3 × 10^2^ pfu group were euthanized over a span of 6 days, which provides limited insights into the tissue tropism of AKMV. Intranasal infection of CAST mice with AKMV led to replication of the virus at the site of inoculation and/or upper respiratory system, then appeared to disseminate to other organs, primarily the lungs and skin. These results, although limited, are similar to what has been observed in other studies with experimentally infected CAST mice [15,16]. Studies have shown CAST and other wild-derived mouse strains to be more susceptible to OPXV infection (*Monkeypox virus*, *Cowpox virus*, *Vaccinia virus*) than other classical inbred strains of mice such as BALB/c [15,16], potentially due to a low level of natural killer cells and a delayed interferon-γ response [18,19]. It remains unclear to what degree the high pathogenicity of AKMV observed in this study is due to the increased susceptibility of CAST mice to OPXVs, or to what degree their susceptibility differs from *Mus musculus* in nature.

The individual tail sample that was PCR-positive (no viable virus) from the negative control group was potentially due to human error leading to DNA contamination during the necropsy of this individual. No other potential DNA contamination issues were observed, and the tissue sampling of the negative control group was conducted independently from other groups.

No viable virus was observed in the oral and anal swabs tested, despite many being PCR-positive, indicating a lack of viral shedding orally or anally following intranasal infection with AKMV in CAST mice. Infection studies with other OPXVs have shown similar results, in which infected animals had viral DNA detected from oral swabs by RT-PCR, but no viral growth in cell culture [20,21]. This might indicate areas where inactivated or attenuated virus is accumulated rather than sites of viral replication [22]. Skin lesions are a major site of replication and viral shedding for most OPXVs and potentially for AKMV as well. AKMV transmission in nature at this point is unknown, however, skin lesions collected from wild caught *Apodemus flavicollis* and *Apodemus uralensis* in the country of Georgia have had a mean viral load of 6 × 10^4^ pfu/mL (range: 4 × 10^1^–1 × 10^5^ pfu/mL) [5].

In our study, relatively high amounts of viable virus were detected in the tail skin and nostril samples collected in the three highest dose groups. Viral skin lesions were not observed; however, the viable virus in the tail samples (80% of high-dose groups—12/15) suggest that AKMV did successfully seed the skin despite the lack of lesions or rash development. This could be a result of the inability of AKMV to cause skin lesions in CAST mice, or perhaps the mice met the pain score criteria for euthanasia prior to development of skin lesions. The high viral load in the nostrils is to be expected due to local spread and replication at the inoculation site.

DNAemia was observed in the two highest dose groups tested and would likely also be present in the AKMV 3 × 10^6^ pfu group if sufficient blood volume had been collected for testing. This, alongside the evidence of viral infection in the skin and other tissues, could indicate that Akhmeta virus is capable of systemic spread throughout this mouse model.

The results of this study identified that a viral load of at least 3 × 10^2^ pfu of AKMV can infect and cause significant disease in CAST mice, which may have implications for the potential role of *Mus musculus* in the natural history of this virus. Human disease caused by exposure to peridomestic rodents (such as *Rattus rattus* or *Mus musculus*) acting as liaison hosts (or ‘bridge hosts’) has been observed with many different zoonotic pathogens, including OPXVs [23,24,25,26,27]. Further field and/or laboratory studies are needed to understand the capability of *Mus musculus* to serve as a liaison host in the country of Georgia. Future AKMV characterization studies should consider evaluating pathogenicity in multiple mouse strains, or perhaps infecting wild-caught mice such as *Apodemus* species, as members of this genus are evidenced to be potential reservoirs of AKMV in Georgia [5].

## Figures and Tables

**Table 1:** Euthanasia criteria for CAST/EiJ mice infected with Akhmeta virus. The highest clinical score within a parameter is assigned, clinical scores are cumulative between parameters, not within. A clinical score of 10 or higher required immediate euthanasia.

Parameter	Clinical Sign	Score
Appearance	Appearance normal	0
	Reduced grooming (dull/rough coat)	1
	Ocular tightening/nasal bulge	2
	Ocular/nasal discharge	3
	Absence of grooming or piloerection	4
	Lesions present in foot sole	4
	Lesions present in mouth	4
	Labored breathing under anesthesia	5
	Labored breathing	8
Body weight	Less than 5% weight loss, or no weight loss	0
	Up to 5% of weight loss	1
	6–15% of weight loss	3
	16–25% of weight loss	5
	>25% of weight loss	10
Behavior	Normal behavior; bright, alert and responsive	0
	Subdued but normal when stimulated	2
	Inappetence	3
	Pronounced cheek bulge/ear and whiskers close to the face	6
	Subdued even when stimulated	8
	Unresponsive when stimulated	10

**Table 2:** Clinical sign observation number of animals per experimental group.

Clinical Sign (CS)	Negative Control	<1 pfu	2 × 10^0^ pfu	3 × 10^2^ pfu	5 × 10^4^ pfu	3 × 10^6^ pfu	CS Totals
Piloerection	0	0	0	5	5	5	15
Weight loss	0	0	3	2	4	5	14
Hunched	0	1	0	0	0	0	1
Labored breathing (LB)	0	0	0	3	0	0	3
LB under anesthesia	0	0	0	1	5	5	11
Dehydration	0	0	0	0	0	5	5
Swollen eye(s)/face	0	0	0	3	5	0	8
Ocular discharge	0	0	0	5	3	0	8
Visible skin lesions	0	0	0	0	0	0	0
Total CS per group:	0	1	3	19	22	20	65
